# When do employees feel isolated when working from home? Longitudinal trajectories, antecedents and outcomes of workplace isolation during the COVID-19 pandemic

**DOI:** 10.3389/fpsyg.2025.1601214

**Published:** 2025-07-08

**Authors:** Ilona Efimov, Annika Krick, Volker Harth, Jörg Felfe, Stefanie Mache

**Affiliations:** ^1^Institute for Occupational and Maritime Medicine, University Medical Center Hamburg-Eppendorf, Hamburg, Germany; ^2^Department of Work, Organizational and Business Psychology, Helmut Schmidt University Hamburg/University of the Federal Armed Forces, Hamburg, Germany

**Keywords:** health-oriented leadership, person-oriented approach, remote work, social relationships at work, teleworking, workplace isolation

## Abstract

**Introduction:**

Previous longitudinal studies investigated loneliness in general populations during the COVID-19 pandemic. Less is known about workplace isolation among employees working from home (WFH). Based on job demands-resources and conservation-of-resources theories, this study aims to analyze workplace isolation of employees WFH in relation to their WFH intensity.

**Methods:**

This study examined the change in workplace isolation and WFH intensity over 5 measurement points of 512 employees using multilevel growth curve analysis (GCA), identified groups of participants with distinct trajectories of workplace isolation and WFH intensity using latent profile analysis (LPA), and investigated antecedents and consequences of profile membership.

**Results:**

GCA indicated an overall negative linear and quadratic relationship between time and workplace isolation, as well as interaction effects between time and WFH intensity on workplace isolation. LPA identified 3 groups: (1) high WFH intensity and low isolation, (2) low WFH intensity and high isolation, (3) high WFH intensity and high isolation. Subsequent analyses revealed that individuals in profile 1 had high levels of health-oriented self-leadership (SelfCare) and social support by colleagues, and low levels of communication difficulties, health-oriented employee-leadership (StaffCare) and extraversion. Regarding differences, highest commitment was identified among individuals displaying low WFH intensity (profile 2), whereas highest self-rated performance was prevalent among individuals experiencing low workplace isolation (profile 1).

**Discussion:**

Applying GCA and LPA in this line of research is novel and adds to the understanding of both between-and within-effects of workplace isolation and WFH intensity. Knowledge about relevant resources (e.g., SelfCare) and demands (e.g., communication difficulties) may inform organizational practices aimed at preventing isolation in remote and hybrid work settings.

## 1 Introduction

The COVID-19 pandemic required worldwide containment and mitigation measures, including social distancing measures, to prevent virus spread (OECD, 2020). These measures negatively impacted loneliness and mental health in general populations across countries (Ernst et al., 2022; Robinson et al., 2022) and specific vulnerable subgroups (Varga et al., 2021), such as older and younger people or those with pre-existing mental illnesses (Varga et al., 2021; Su et al., 2023). Research has clearly demonstrated a negative impact of loneliness and social isolation on physical and mental health outcomes, both before (Leigh-Hunt et al., 2017) and during the pandemic (Loades et al., 2020; Xiong et al., 2020).

Social distancing also brought significant changes to workplaces, including the introduction or increase of remote, digital collaboration (Hernandez and Abigail, 2020). This paper uses the term “work from home” (WFH) to focus on employees working from home (De Vincenzi et al., 2022). But not all organizations were technically and organizationally well prepared for this change during the pandemic (Wang et al., 2021; Efimov et al., 2024a). Current research investigates WFH’s effects on employee outcomes, as this relationship is far from univocal. A comprehensive meta-analysis found overall small but beneficial effects of remote work intensity on employee outcomes, although perceived autonomy had a reinforcing and isolation perceptions a detrimental effect as mediators in this relationship (Gajendran et al., 2024). Further, a large-scale study identified a nonlinear relationship between WFH intensity and professional isolation, demonstrating a “too much of a good thing” effect (Rudolph and Zacher, 2024). WFH intensity describes “the extent to which individuals work virtually, away from the office” (Golden et al., 2008, p. 1413). Past research on WFH outcomes suggested that the amount of time people WFH may be more relevant than whether or not they actually do WFH (Rudolph and Zacher, 2024). This study (Rudolph and Zacher, 2024) and reviews (Wang et al., 2021; Beckel and Fisher, 2022; De Vincenzi et al., 2022) suggested that personal and organizational factors influence employees’ perceptions of remote work, rather than this working arrangement having a homogenous impact on employees (De Vincenzi et al., 2022).

Despite a considerable increase in remote collaboration in recent years, workplace-related isolation remains under-researched (Sahai et al., 2020). Workplace isolation is “a two-dimensional construct that represents individuals’ perception of isolation from others at work and includes perceived isolation from both colleagues and from the company’s support network” (Marshall et al., 2007, p. 195). The first dimension “colleagues” therefore describes the perceived isolation from colleagues, i.e., when the need for social or emotional interaction or camaraderie at work is not fulfilled. The second dimension “company” describes the perceived isolation from the company, i.e., when employees do not feel part of the team or departmental network (thus do not get help and support in specific work-related needs) and that their achievements are not being acknowledged by the company. The subjective perception of isolation and not necessarily the actual physical proximity is decisive for this construct (Marshall et al., 2007). Accordingly, workplace isolation is a specific construct that only applies to the work context. In this study, the focus will be on the first dimension of this construct: perceived isolation from colleagues at work. Workplace isolation is distinct from loneliness, a broader, emotional construct that can be applied to all population groups (Russell et al., 1984; Marshall et al., 2007). Loneliness refers to “individual’s subjective perception of deficiencies in his or her network of social relationships” whereby these deficiencies can be both qualitative and quantitative in nature (Russell et al., 1984, p. 1313).

In summary, while the pandemic exacerbated loneliness in general populations worldwide, little is known about the temporal dynamics of workplace isolation in relation to WFH intensity over time (trajectories over time) as well as about whether and how subgroups of employees in these trajectories differ (trajectory profiles). Overall, this study aims to analyze workplace isolation among employees working from home in relation to their WFH intensity, identifying a general trend over time and subgroups with different trajectories. Furthermore, this study aims to determine antecedents and consequences of these subgroups with different trajectories.

On the one hand, this study provides theoretical contributions by applying a novel methodological approach to research workplace isolation, enhancing the understanding of both between-and within-person effects, thereby highlighting heterogeneity among employees’ experiences and responses to WFH. It integrates Job Demands-Resources (JD-R) and Conservation-of-Resources (COR) theories to advance the understanding of how individual and social resources and demands influence workplace isolation, thus providing a more comprehensive theoretical framework for studying WFH dynamics. On the other hand, this study offers practical contributions by informing organizations about distinct employee subgroups that may experience workplace isolation differently when WFH and identifies relevant resources and demands in this relationship. This knowledge enables organizations to tailor more effective and personalized support strategies and develop targeted, long-term interventions to mitigate workplace isolation and enhancing employee outcomes, also for the post-pandemic context.

### 1.1 A variable-oriented approach: multilevel growth curve analysis for changes in workplace isolation and WFH intensity

Current state of research demonstrated that longitudinal analyses of changes in isolation perceptions during the pandemic have generally focused on loneliness in the adult general population rather than work-related isolation. These studies were conducted at different time points: before and during the pandemic (Bu et al., 2020; Entringer and Kröger, 2020; Hettich et al., 2022), at the beginning (Buecker et al., 2020; Killgore et al., 2020; Luchetti et al., 2020), and in the course of the pandemic (Caro et al., 2022; Benke et al., 2023; Weber et al., 2023). The methodology and results of studies were mixed: most reported an increase in loneliness (e.g., Hettich et al., 2022; Benke et al., 2023), whilst others found both increases and decreases (e.g., Bu et al., 2020; Buecker et al., 2020), and some noted a decrease (Weber et al., 2023) or stable values (Caro et al., 2022). None of those studies examined slopes of trajectories of isolation perceptions or loneliness over time. However, these studies focused on identifying vulnerable groups and risk factors.

To identify a general trend of workplace isolation and WFH intensity over time, a variable-oriented approach was applied (Laursen and Hoff, 2006). It is appropriate for investigating interindividual variation (Bergman and Lundh, 2015). Yet, there are no existing studies on growth curves of employees’ workplace isolation when WFH (e.g., linear, quadratic or cubic) in general nor during pandemic. This study aims to examine an averaged trajectory of workplace isolation in relation to WFH intensity across participants over time. It is conceivable that employees feel less isolated when WFH over time due to improvements in digital communication tools. Organizations have increasingly invested in better and more diverse digital communication platforms, enhancing collaboration and social interaction (Efimov et al., 2024b). These tools might have improved communication effectiveness, reducing feelings of isolation. Furthermore, employees and leaders have adapted to new work practices, fostering routines that promote social interaction and collaboration even in a remote environment (Efimov et al., 2024a). The initial uncertainty and discomfort in handling WFH might have diminished over time, reducing workplace isolation. Organizations have started organizing targeted virtual social activities and team-building events to strengthen the sense of community and combat isolation, encouraging informal exchanges and social interactions (Efimov et al., 2024a). Thus, we assume:

*H1a*: There is a negative nonlinear relationship between time and workplace isolation.

Many organizations have implemented hybrid work models, combining WFH with office presence. These models offer employees flexibility to WFH while also providing opportunities for in-person interactions at the office. However, increased WFH intensity reduces direct, personal interactions with colleagues, potentially exacerbating workplace isolation. Thus, the loss of spontaneous communication and informal exchanges that occur in the office (Tautz et al., 2022; Krick et al., 2023; Efimov et al., 2024a) can potentially increase feelings of isolation. High WFH intensity may impair team dynamics, as employees have fewer opportunities to build and maintain team relationships and trust (Breuer et al., 2016), further potentially increasing isolation. Despite the availability of digital communication tools, technological issues and the lack of personal proximity can degrade interaction quality (Efimov et al., 2024a; Klebe et al., 2024), enhancing isolation with higher WFH intensity. Given this, it is conceivable that workplace isolation and WFH intensity covary over time (e.g., Gajendran et al., 2024; Rudolph and Zacher, 2024) and that time and WFH intensity interact with each other, which means that the trajectory of WFH intensity predicts how workplace isolation develops over time. However, no studies to date considered WFH intensity as influencing variable in the relationship between time and workplace isolation. Past research so far indicated that WFH intensity may serve as a predictor or moderator of perceptions of isolation in remote collaboration (Golden et al., 2008; Sahai et al., 2020). A recent meta-analysis found a mediating effect of isolation between remote work intensity and employee outcomes (Gajendran et al., 2024). Moreover, a large-scale longitudinal study indicated a nonlinear (“u-shaped”) relationship between WFH intensity and professional isolation, indicating a “too much of a good thing” (Rudolph and Zacher, 2024). Thus, we assume:

*H1b*: There is an interaction effect of time and WFH intensity on workplace isolation. The trajectory of WFH intensity predicts workplace isolation over time.

### 1.2 A person-oriented approach: distinct trajectories of workplace isolation and WFH intensity development

Since people may differ in their trajectories, a person-oriented approach was applied. This approach is appropriate for studying individual development and identifying subgroups with different trajectories. It is characterized by a pattern focus, an individual focus, and a process focus. Thereby, the person-oriented approach is theoretically and methodologically opposed to the variable-oriented approach (Bergman and Lundh, 2015).

To date, there are only a few longitudinal studies on distinct trajectories of loneliness or isolation perceptions during the pandemic. Three studies on loneliness revealed four subgroups of loneliness trajectories, but heterogenous results on trajectory prevalence, slopes and levels: Each study found at least one subgroup with high, medium and low levels of loneliness (Bu et al., 2020; Laham et al., 2021; Caro et al., 2022). Another study examined distinct trajectories of workplace sense of community of employees WFH and identified two latent classes of high and low trajectories (Graham et al., 2023). However, this construct is distinct from workplace isolation.

No studies have yet explored distinct trajectories of both workplace isolation and WFH intensity. However, existing studies on associations between workplace isolation and WFH intensity (Golden et al., 2008; Sahai et al., 2020; Gajendran et al., 2024; Rudolph and Zacher, 2024) suggest a covariation over time. Further, we assume WFH intensity to be a relevant variable for identifying subgroups regarding workplace isolation perceptions. To date, latent profile analysis (LPA), a method of the person-oriented approach, has been used little in vocational behavior research (Spurk et al., 2020), and even more rarely have two variables been considered in an LPA (e.g., Kinnunen et al., 2019).

Applying a person-oriented approach (Laursen and Hoff, 2006), this study aims to identify subgroups (profiles) with distinct trajectories of workplace isolation in relation to WFH intensity over time. Due to the limited, heterogeneous findings of past research, we expect at least two levels of both workplace isolation and WFH intensity (i.e., high and low), resulting in four groups: (1) high workplace isolation, high WFH intensity, (2) high workplace isolation, low WFH intensity, (3) low workplace isolation, low WFH intensity, (4) low workplace isolation, high WFH intensity. Thus, we expect at least 4 profiles based on these two dimensions (workplace isolation and WFH intensity), which correspond to a four-field scheme. Based on current research, no detailed expectations are set regarding the prevalence or slopes of subgroups. Thus, we assume:

*H2*: Quantitatively and qualitatively distinct trajectories of workplace isolation and WFH intensity can be identified: two trajectories displaying high workplace isolation, one at low and one at high WFH intensity (H2a), and two trajectories displaying low workplace isolation, one at low and one at high WFH intensity (H2b).

### 1.3 Antecedents of profile membership: individual and social determinants

To understand the factors affecting profile membership, the JD-R and COR theories were applied as theoretical framework for this study (Bakker and Demerouti, 2007; Hobfoll et al., 2018). On the one hand, the JD-R theory assumes that each occupation has specific job demands (which, if high, can impair the mental and physical health of employees) and job resources (which, if high, can have positive effects on work-and health-related outcomes and can mitigate the negative effects of job demands; Bakker and Demerouti, 2007). On the other hand, the COR theory assumes that “individuals strive to obtain, retain, foster, and protect those things they centrally value” (Hobfoll et al., 2018, p. 2). Accordingly, this motivational theory posits that people draw on key resources to respond to stress and develop a reservoir of sustaining resources for future needs. The associated resource loss spiral in this theory further assumes that people experience stress when resources are exhausted, and few resources remain to reduce stress. Consequently, further resources are lost in each iteration of the stress spiral to cope with stress. The same applies vice versa for the resource gain spiral, although slower and smaller in magnitude. Yet, gaining resources is essential to prevent future resource losses. People therefore invest in resources in order to protect and gain resources (Hobfoll et al., 2018). There are various approaches for investigating the effects of working conditions on employee outcomes when WFH. Following Wang et al. (2021), this study also considers work characteristics as antecedents in the WFH context. Since the meaning of work characteristics may be shaped by context and WFH became the “new normal”, specific work characteristics should be examined. This allows an investigation of which work characteristics act as demands or resources for different subgroups regarding workplace isolation and WFH intensity. With respect to investigating antecedents of profile membership as another aim of our study, we consider facilitating and hindering factors (both demands and resources) on different levels: individual and social determinants at work.

In terms of *individual determinants*, we examine employees’ health-oriented self-leadership (SelfCare) and extraversion as predictors of profile membership. According to Health-oriented Leadership (HoL) model (Franke et al., 2014), SelfCare describes how employees manage their own health. In addition to employees’ SelfCare, the HoL model comprises two further components: managers’ SelfCare and StaffCare (i.e., health-oriented employee-leadership). Each of the components consists of three dimensions: value, awareness, and behavior. In terms of employee SelfCare, these three dimensions imply that employees can lead themselves in a health-oriented manner if they care about their health (value), are aware of changes in their state of health (awareness) and can apply appropriate health-promoting practices (behavior). The model assumes that positive characteristics of all three components improve the well-being and health of the workforce (Franke et al., 2014). A German longitudinal study during pandemic showed that SelfCare was more prevalent among employees when WFH, and that WFH intensity demonstrated a moderating (i.e., intensifying) effect on the effectiveness of SelfCare on employees outcomes (less strain and health complaints as well as more relaxation and performance; Krick et al., 2024a). Thus, it is considered a personal resource that, according to COR theory, can facilitate further resource gain and reduce resource depletion, which in turn may decrease perceptions of workplace isolation when WFH.

In addition, employees’ personality traits may influence workplace isolation perceptions when WFH. Extraversion, a Big Five personality trait associated with higher engagement in social activities (Lucas et al., 2008), is a relevant, rather time-stable predictor in this study that may increase perceptions of workplace isolation when WFH. Previous research has pointed to a negative link between extraversion and preference for teleworking (Gavoille and Hazans, 2022). A study with police officers WFH during lockdown found that individuals with higher extraversion scores missed their colleagues more (Langvik et al., 2021). Further, a literature review argued that new ways of working may be stressful for highly extraverted individuals, since they miss face-to-face contact with colleagues more (Demerouti et al., 2014). Thus, we assume:

*H3*: Individual determinants (SelfCare, extraversion) predict profile membership. SelfCare (H3a) is positively, and extraversion (H3b) is negatively associated with trajectory profiles which indicate low workplace isolation and high WFH intensity.

In terms of *social determinants*, we examine social norm, social support by colleagues, health-oriented employee-leadership (StaffCare; Franke et al., 2014), and communication difficulties as predictors of profile membership. For one, we expect that social norm at work, i.e., the extent of how many colleagues WFH, may mitigate workplace isolation. Past research indicated that social influence affects decision making, e.g., choice to telework, and that teleworking colleagues had a stronger influence on telework adoption than non-teleworking colleagues (Scott et al., 2012). Thus, a high number of colleagues WFH might positively influence WFH experiences, i.e., employees may feel connected in their experience and perceive a strengthened community that is well attuned to remote collaboration.

Moreover, we expect social support by colleagues to decrease perceptions of workplace isolation when WFH (Marshall et al., 2007; Sahai et al., 2020). Social support at work may be crucial in reducing perceived workplace isolation as it fosters a sense of belonging to a team or organization, both face-to-face and when WFH (Marshall et al., 2007; Graham et al., 2023). Knowing that colleagues and supervisors are available for assistance may help mitigate stress and challenges, making employees feel less isolated. Emotional support from colleagues or supervisors can alleviate negative emotions such as loneliness or frustration (Marshall et al., 2007). Social support also provides access to professional resources (e.g., knowledge and feedback; Drageset, 2021; Jolly et al., 2021), enhancing integration and reducing isolation, creating a more inclusive and interconnected work environment where employees feel more engaged (Marshall et al., 2007; Sahai et al., 2020).

Likewise, according to the HoL model (Franke et al., 2014), we also expect StaffCare, i.e., leader’s care for followers health, to be another inhibiting factor for workplace isolation when WFH. Past research suggested a negative link between leadership in remote collaboration and perceptions of isolation (Efimov et al., 2022) and identified leadership as antecedent of workplace isolation (Sahai et al., 2020). Although there are currently no studies on the link between StaffCare and employee isolation when WFH, previous studies indicated a beneficial effect of StaffCare on employee outcomes when WFH (Klebe and Felfe, 2023; Krick et al., 2023; Sachse and Schanz, 2023) or in times of crisis (Klebe et al., 2021). Still, its effectiveness may be impaired by information and communication technologies (ICT) hassles (Klebe et al., 2023). Leaders actively taking care of their employees’ health in terms of StaffCare should reduce workplace isolation by fostering interaction and engagement through regular communication (Henke et al., 2022; Efimov et al., 2024a). This emotional support may mitigate loneliness and foster a sense of belonging. Leaders modelling health-oriented behavior create a supportive workplace culture that encourages peer support, reduces stress and depression (Franke et al., 2014), and may diminish isolation. Providing resources for social interaction, such as team-building activities, allows employees to connect with each other, further reducing isolation (Efimov et al., 2024a).

Last, we consider communication difficulties during WFH as a conducive factor for workplace isolation. Previous studies demonstrated that electronic communication may lack richness and social presence (Marshall et al., 2007), and that lack of social presence, limited informal chats and communication difficulties were experienced as challenges by employees in remote setting compared to traditional office setting (Tautz et al., 2022). Further studies illustrated that communication quantity and improved access to ICT were able to reduce isolation perceptions in remote work (Van Zoonen and Sivunen, 2022; Wong et al., 2022) and decrease professional isolation’s impact on teleworkers’ work outcomes (Golden et al., 2008). Thus, we assume:

*H4*: Social determinants (social norm, social support by colleagues, StaffCare, communication difficulties) predict profile membership. Social norm, social support by colleagues, and StaffCare are positively associated with trajectory profiles which indicate low workplace isolation and high WFH intensity (H4a). Communication difficulties are negatively associated with trajectory profiles which indicate low workplace isolation and high WFH intensity (H4b).

### 1.4 Consequences of profile membership: health-and work-related outcomes

Studying consequences of distinct trajectories of workplace isolation and WFH intensity is highly relevant. Following the COR theory (Hobfoll et al., 2018), we assume that employees experiencing lower levels of workplace isolation have more resources and, according to resource gain spiral, that this is linked to positive health-and work-related outcomes. Previous research indicated a positive link between workplace isolation and burnout or emotional exhaustion, and a negative link to affective commitment and job performance (Sahai et al., 2020). Considering WFH intensity, a longitudinal study during the pandemic pointed to an indirect effect of remote work on psychological distress via isolation (Van Zoonen and Sivunen, 2022). Most recently, a meta-analysis found an overall positive relationship between remote work intensity and various employee outcomes (e.g., organizational commitment and supervisor-rated job performance), with isolation perceptions having a detrimental effect as mediator (Gajendran et al., 2024). To date, studies have not used a person-oriented approach to gain differentiated insights into the consequences (health and work-related outcomes) of subgroups with distinct trajectories in terms of workplace isolation perceptions and WFH intensity. This study aims to address this gap by examining differences between identified profiles in terms of one health-related outcome (psychological strain) and two work-related outcomes (commitment, self-rated performance).

*H5*: Trajectory profiles differ in their levels of psychological strain, commitment, and self-rated performance. Trajectory profiles which indicate low workplace isolation and high WFH intensity display lower levels of psychological strain (H5a), and higher levels of commitment (H5b), and self-rated performance (H5c).

**Figure 1:**
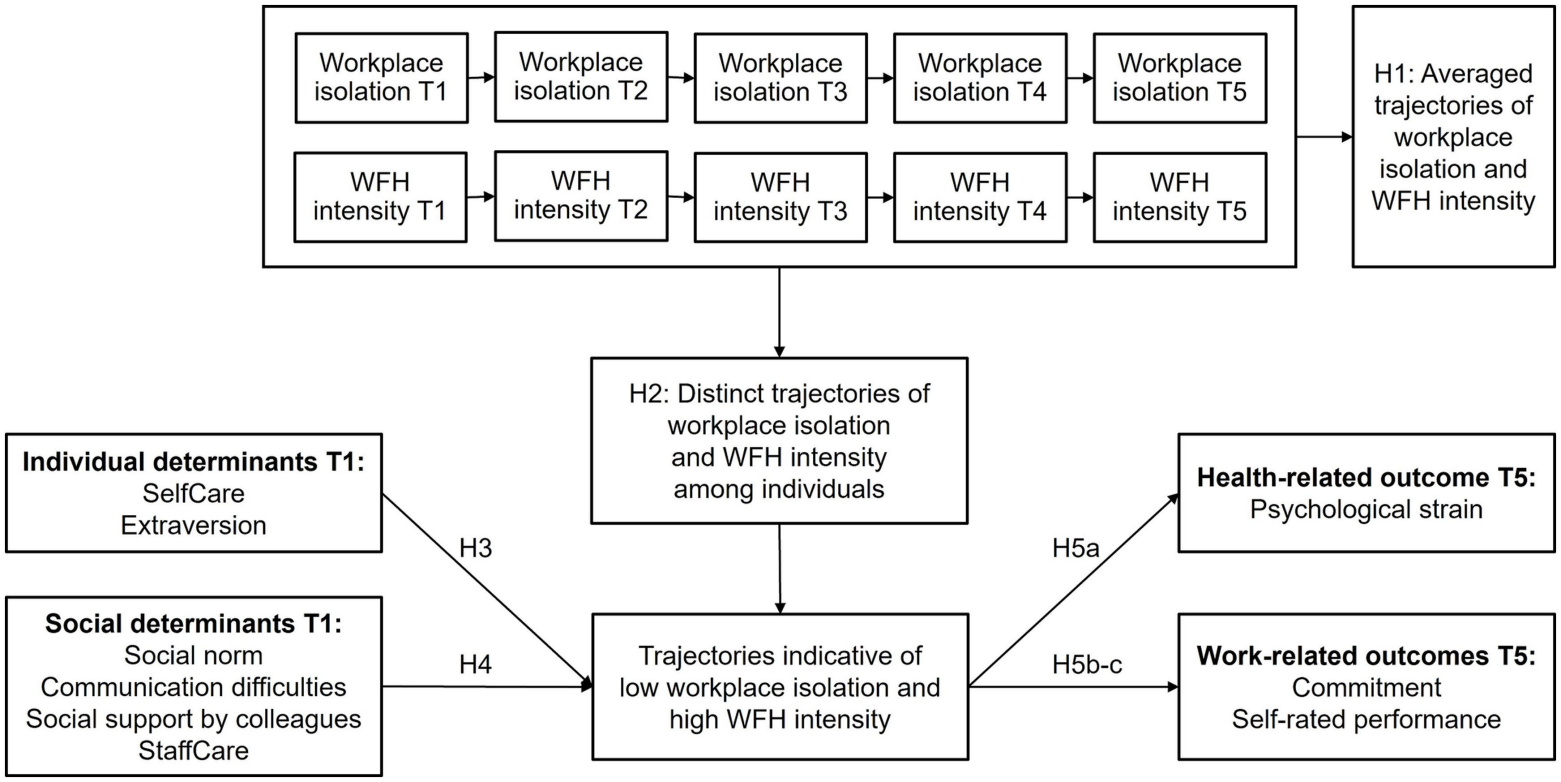
Proposed model.

See Figure 1 for our proposed research model (oriented on the structure of model by Kröner and Müller, 2023).

## 2 Method

### 2.1 Study design and sample

We applied a multilevel mixed design by analyzing five measurement points (level 1) nested within *N* = 512 individuals (level 2). Data was collected in Germany by a market research institute via an online survey in 5 waves. The T1 survey included measures of sociodemographic and job-related variables, personal resources, job demands and resources (as potential predictors of latent profile membership). All surveys (T1-T5) included measures of workplace isolation and WFH intensity. Outcome variables (i.e., psychological strain, commitment and self-rated performance) were measured at T5. Eligibility criteria for participation was full-time employment, which generally allowed WFH. Part-time employees, self-employed persons (without employees), unemployed persons, pensioners, housewives and-men, full-time students and participants who were on short-time work or temporary work were excluded. Participation was voluntary and all study participants received written information about the study beforehand and signed an informed consent form regarding data collection and analysis. The study was conducted in accordance with the Declaration of Helsinki and adheres to the legal requirements of the study country.

A total of 848 questionnaires were completed at 5 measurement points by participants with varying degrees of WFH intensity (from 0 to 5 days per week WFH). To analyze a sample that worked for at least one day per week from home at all measurement points, the data set was reduced to a final sample size of *n* = 512 participants. Thus, study participants worked either hybrid or fully remote. They were on average 48.56 years old (*SD* = 10.96) and half of them were female (49.8%). At the time of the survey, all participants worked full-time in different sectors. At T1, almost half worked from home 5 days a week (46.9%) and in companies with more than 500 employees (50.4%). Most held no leadership role (62.5%), were in a salaried employment (86.5%) and had no responsibility for childcare while WFH (65.2%). Further information on sample characteristics is presented in Table 1.

**Table 1:** Sociodemographic and job-related characteristics of the sample.

Variables	*n*	%
Gender	512	
Female	255	49.8
Male	257	50.2
Age*	512	
18–29 years	33	6.4
30–39 years	75	14.6
40–49 years	133	26.0
50–59 years	169	33.0
60–69 years	102	19.9
Highest level of education	510	
High School	153	29.9
Vocational training	140	27.3
University degree	196	38.3
PhD	21	4.1
Household members	397	
Partner	346	67.6
Children under 5 years of age	39	7.6
Children between 6 and 14 years of age	90	17.6
Children over 14 years of age	102	19.9
(Grand-) Parents	9	1.8
Others (e.g., roommates)	10	2.0
Care for (grand) children while WFH	512	
Never	334	65.2
Rarely	36	7.0
Sometimes	67	13.1
Often	52	10.2
Almost always	23	4.5
Employment	512	
Salaried employment	443	86.5
Civil servant	36	7.0
Self-employed	33	6.4
Company size	512	
≤10 employees	46	9.0
11–49 employees	46	9.0
50–99 employees	43	8.4
100–500 employees	119	23.2
≥ 500 employees	258	50.4
Leadership role	512	
No	320	62.5
Yes	192	37.5
WFH intensity	512	
Up to 1 day per week	21	4.1
2 days per week	73	14.3
3 days per week	94	18.4
4 days per week	84	16.4
5 days per week	240	46.9
Industry	512	
Metal and electrical industry	40	7.8
Chemical and pharmaceutical industry	18	3.5
Energy industry	8	1.6
Construction industry	17	3.3
Craft industry	9	1.8
Logistics, transportation and traffic	25	4.9
Tourism, hotels and restaurants	11	2.1
Banking and insurance industry	57	11.1
Real estate, property & rental industry	9	1.8
Consulting (Management, legal, personnel, tax)	20	3.9
Advertising, communication, marketing, market research, PR	4	0.8
Trade industry	43	8.4
Security	2	0.4
IT, telecommunications	69	13.5
Education, childcare and science	26	5.1
Care, medicine and health	16	3.1
Media, art and culture	7	1.4
Public administration	81	15.8
Other	50	9.8

*M = 48.56, *SD* = 10.96, range = 19–66 years. All variables were measured at T1.

### 2.2 Measures

#### 2.2.1 Main variables

*Time* of the survey was coded with T1-T5: The first wave started in April 2021 (T1), continued in July 2021 (T2), September 2021 (T3), July 2022 (T4), and ended in October 2022 (T5).

To assess perceived *workplace isolation* when WFH, we adapted three items of the professional isolation scale by Golden et al. (2008): “I miss face-to-face contact with coworkers”, “I feel isolated”, and “I miss informal interaction with others” with answer options ranging from 1 = *Does not apply at all* to 5 = *Totally applies*. Cronbach’s *α* for T1 was 0.84, T2: α = 0.85, T3: α = 0.82, T4: α = 0.83 and for T5: α = 0.84.

*WFH intensity* was measured at T1 to T5 using a single self-constructed item, oriented on the single item developed by Golden et al. (2008): “On average, how often did you work from home in the last 4 weeks?” on a 5-point scale including: 1 = *maximum 1 day per week*, 2 = *2 days per week*, 3 = *3 days per week*, 4 = *4 days per week* to 5 = *at least 5 days per week*.

#### 2.2.2 Predictors of profile membership

All predictors used in the analyses were measured at T1 with answer options ranging from 1 = *Does not apply at all* to 5 = *Totally applies* (except for items on communication difficulties).

*SelfCare* (leader’s and followers’ care for their own health) was measured by using 16 items of the HoL scale by Franke et al. (2014): four items on health awareness, e.g., “I immediately notice when something is wrong with my health.” or “I often notice too late when I am expecting too much of myself.”, one item on value of health: “My health is my first priority.”, and 11 items on health behavior, e.g., “I try to reduce my demands by optimizing my personal work routine (e.g., set priorities, care for undisturbed working, daily planning).” or “When things have been stressful for a longer amount of time, I make sure to slow down afterwards.” Cronbach’s *α* was 0.79.

*Extraversion* was assessed by using three items of the Big Five Inventory-SOEP by Schupp and Gerlitz (2008): “I see myself as someone who is talkative.”, “I see myself as someone who is reserved, quiet.” (recoded) and “I see myself as someone who is outgoing, sociable.” Cronbach’s *α* was 0.74.

*Social norm* regarding WFH was assessed by using one single self-constructed item: “Most of my colleagues and employees in my department work mainly from home.”

*Social support by colleagues* when WFH was surveyed by using one item of Eurofound’s 6th European Working Conditions Survey (Eurofund, 2015): “My colleagues help and support me.”

*StaffCare* (follower-rating of the leader’s care for followers health) was assessed by using 19 items of the HoL scale by Franke et al. (2014): four items on health awareness, e.g., “My supervisor immediately notices when something is wrong with my health.” or “My supervisor does often not notice when he/she asks too much of me.”, one item on value of health: “My health is important to my supervisor.”, and 14 items on health behavior, e.g., “When I seem to be stressed, my supervisor responds to it and tries to propose solutions.” or “My supervisor tries to reduce my demands by optimizing my work-life balance (e.g., take regular breaks, avoid overtime, avoid the expiration of vacation days).” Cronbach’s α was 0.89.

*Communication difficulties* when WFH were measured by using three self-constructed items: “Communication with others (team members, supervisors, clients) is complicated and inconvenient.”, “There are misunderstandings in communication/agreements with others.”, “There are uncertainties in communication/agreements.” with answer options ranging from 1 = *Never* to 5 = *Almost always*. Cronbach’s α was 0.88.

We controlled for age, gender, care for (grand) children while WFH and voluntariness of WFH as these variables may influence analyses on workplace isolation and WFH intensity (Kaduk et al., 2019; Rieth and Hagemann, 2021; Vanderstukken et al., 2022). We assessed care for (grand) children while WFH by using one item: “To what extent do you care for children or grandchildren while working from home?” with answer options ranging from 1 = *Never* to 5 = *Almost always*. Voluntariness of WFH was measured with one item (modified from Kaduk et al., 2019): “I can decide whether and how often I work from home.” with answer options ranging from 1 = *Does not apply at all* to 5 = *Totally applies*.

#### 2.2.3 Outcomes of profile membership

All outcome variables used in the analyses were measured at T5.

*Psychological strain* was assessed using four items of the irritation scale by Mohr et al. (2005): “I have difficulty relaxing after work.”, “I get grumpy when others approach me.”, “I anger quickly.”, and “I get irritated easily, although I do not want this to happen.” with answer options ranging from 1 = *Never* to 5 = *Almost always*. Cronbach’s α was 0.89.

*Affective organizational commitment* was measured using four items of the COMMIT scale by Felfe and Franke (Schilling, 2014): “I feel a strong emotional connection to my organization.”, “I am proud to be part of my organization.”, “I feel a strong sense of belonging to my organization.” and “I think that my values match those of my organization.” Items were rated on a 5-point scale: 1 = *Does not apply at all* to 5 = *Totally applies*. Cronbach’s α was 0.95.

*Self-rated performance* when WFH was measured using a self-constructed item: “Based on the last 4 weeks, how would you rate your overall work performance when working from home (in terms of effectiveness and productivity)?” on a 5-point scale: 1 = *sufficient* to 5 = *excellent*.

See Supplementary material 1 for an overview of variables used in this longitudinal study. The questionnaire will be made available on reasonable request by the authors.

### 2.3 Data analysis

In order to model the rate of changes of workplace isolation over time, we conducted a multilevel growth curve analysis (GCA) in IBM® SPSS® Statistics (version 26, IBM, Armonk, NY, USA) by following recommendations by Field (2013). This analysis method is classified as a multilevel mixed effects model. As a method of the variable-oriented approach, it is appropriate for investigating interindividual variation over time (Bergman and Lundh, 2015). Additionally, we examined whether WFH intensity interacts with time in predicting workplace isolation. Therefore, we restructured our data into long format and checked for outliers, normal distribution of residuals using skewness, kurtosis as well as histograms and Q-Q plots, multicollinearity, and heteroscedasticity using scatterplots. For GCA, we first modelled a linear relationship (model 1) between time (i.e., measurement point 1 to measurement point 5) and our dependent variable workplace isolation and added an interaction term between time and WFH intensity. We then continued with a quadratic model (model 2) and a cubic relationship (model 3). For all analyses we processed with raw data, no centering of predictors. We used “AR(1): Heterogeneous” (i.e., first-order autoregressive structure, meaning that relationship between variances changes in a systematic way; Field, 2013) as covariance structure since we had repeated measures over time, and we assumed that values were less correlated over time and variances were heterogenous, and we identified better model fit indices in comparison to “AR(1).” Further, we used maximum likelihood for model estimation to compare models, and as it also provides more accurate estimates of fixed parameters. For evaluating which model best describes the rate of changes of workplace isolation over time, we tested differences in-2LL (log-likelihood) using chi-square statistics. The model with the smallest, significant -2LL value best describes the data (Field, 2013).

Adopting a person-oriented approach, latent profile analysis (LPA) was used to identify distinct (latent) profiles based on the levels and changes of both workplace isolation and WFH intensity over the course of the pandemic. LPA is a type of finite mixture modelling and combines growth curve modelling and latent class analysis. Thus, it identifies latent classes and calculates parameter estimates for each latent class (Muthén and Muthén, 1998-2015). Latent classes refer to clustering of individuals to latent subgroups. To date, the method LPA has been used little in vocational behavior research (Spurk et al., 2020), and even more rarely were two variables considered in an LPA. We have oriented our research on the study by Kinnunen et al. (2019). Following recommendations of a systematic review on LPA in vocational behavior research (Spurk et al., 2020), we considered our sample size to be sufficient for the calculation of an LPA. By defining explicit hypotheses about the number of profiles, a confirmatory LPA (in contrast to a fully exploratory LPA) was performed in order to identify latent subgroups (Muthén and Muthén, 1998-2015; Spurk et al., 2020). We validated our profiles in subsequent analyses by testing hypotheses on predictors on profile membership as well as on mean differences across profiles in relation to theoretically relevant outcomes (Spurk et al., 2020). We used maximum likelihood with robust standard errors and treated WFH intensity and workplace isolation as continuous variables (Muthén and Muthén, 1998-2015). For selecting the best fitting profile solution, past research recommended using multiple statistical fit values as well as considering content decision criteria (Spurk et al., 2020). When comparing different profile solutions, we chose the model with the smallest Bayesian information criterion (BIC) value, significant *p*-values in Lo–Mendell–Rubin likelihood ratio test (LMRT), Vuong-Lo–Mendell–Rubin likelihood ratio test (VLMRT), and the bootstrapped likelihood ratio test (BLRT), as well as the classification quality based on entropy (i.e., overall accuracy of classification) and average latent class posterior probabilities (AvePP, i.e., the certainty of sorting a person into a specific class; Spurk et al., 2020). Additionally, we considered the trajectory prevalence (according to rule of thumb: minimum of 1% or > 25 cases per profile; Spurk et al., 2020) and the interpretability of the trajectories for our decision. The analysis was performed using Mplus 6.12 (Muthén and Muthén, 1998-2015).

Once a profile solution was identified, we performed a multinomial logistic regression using IBM® SPSS® Statistics to examine predictors of profile membership. We checked our data for linearity of the logit, independence of errors (overdispersion), multicollinearity, outliers, incomplete separation, and analyzed main effects. Profile 1 “high WFH intensity and low isolation” was defined as reference category.

The last step in the analysis involved performing univariate analyses of variance (ANOVAs) with post hoc tests using IBM® SPSS® Statistics to examine differences between profile trajectories on outcomes. We checked our data for homogeneity of variance, normal distribution, and outliers within groups.

## 3 Results

Means, standard deviations, and correlations are presented in Table 2.

**Table 2:** Means, standard deviations, and correlations.

Variables	*M*	*SD*	1	2	3	4	5	6	7	8
1. Workplace isolation T1	3.22	1.13								
2. Workplace isolation T2	3.05	1.13	0.69**							
3. Workplace isolation T3	2.93	1.06	0.68**	0.70**						
4. Workplace isolation T4	2.78	1.09	0.63**	0.62**	0.66**					
5. Workplace isolation T5	2.81	1.08	0.64**	0.65**	0.64**	0.75**				
6. Gender^a^	/	/	−0.00	−0.01	−0.05	−0.04	−0.09*			
7. Age^a^	48.56	10.96	−0.09	−0.05	−0.07	−0.07	0.00	−0.16**		
8. Care for (grand) children while WFH^a^	1.82	1.25	0.08	0.07	0.11*	0.10*	0.11*	−0.03	−0.20**	
9. Voluntariness of WFH^a^	3.46	1.36	0.03	0.03	0.07	0.07	0.03	−0.09*	0.02	0.07
10. SelfCare^a^	3.45	0.56	−0.19**	−0.11*	−0.11*	−0.16**	−0.14**	−0.01	0.06	−0.10*
11. Extraversion^a^	3.53	0.85	0.17**	0.15**	0.16**	0.09*	0.09	0.10*	0.08	0.09
12. Social norm^a^	3.74	1.24	0.10*	0.09*	0.12**	0.07	0.03	−0.05	−0.06	−0.00
13. Social support by colleagues^a^	3.70	1.02	−0.07	−0.07	−0.06	−0.09*	−0.12**	0.01	−0.02	−0.03
14. StaffCare^a^	2.58	0.79	0.06	0.06	0.08	0.08	0.06	−0.12*	−0.09	−0.00
15. Communication difficulties^a^	2.35	0.88	0.35**	0.29**	0.24**	0.30**	0.30**	−0.06	−0.08	0.22**
16. Psychological strain^b^	2.32	0.90	0.13**	0.12**	0.05	0.15**	0.14**	0.12**	−0.12**	0.05
17. Commitment^b^	3.37	1.06	0.00	0.04	0.03	0.05	0.05	−0.03	0.15**	0.06
18. Self-rated performance^b^	3.56	0.85	−0.21**	−0.17**	−0.19**	−0.24**	−0.26**	0.05	0.10*	−0.04

Variables	9	10	11	12	13	14	15	16	17	18
9. Voluntariness of WFH^a^										
10. SelfCare^a^	0.10*									
11. Extraversion^a^	0.14**	0.16**								
12. Social norm^a^	0.13**	0.05	−0.08							
13. Social support by colleagues^a^	0.16**	0.26**	0.10*	0.07						
14. StaffCare^a^	0.10	0.36**	0.09	−0.01	0.22**					
15. Communication difficulties^a^	−0.10*	−0.29**	−0.11*	0.03	−0.22**	−0.16**				
16. Psychological strain^b^	0.01	−0.42**	−0.16**	−0.02	−0.13**	−0.18**	0.32**			
17. Commitment^b^	0.15**	0.25**	0.21**	−0.10*	0.10*	0.39**	−0.14**	−0.24**		
18. Self-rated performance^b^	0.12**	0.30**	0.05	0.02	0.14**	0.14*	−0.27**	−0.28**	0.25**	

*N* = 512 (only for StaffCare: *n* = 320, Extraversion: *n* = 510). Two-tailed Pearson correlation coefficients were used. StaffCare = health-oriented employee leadership, SelfCare = health-oriented self-leadership, WFH = work from home. T1-T5 = measurement point 1–5. ^a^ = control and predictor variables measured at T1; ^b^ = outcome variables measured at T5. Gender is coded 0 = male and 1 = female. *p < 0.05, **p < 0.01, ***p < 0.001.

### 3.1 Multilevel growth curve analysis for changes in workplace isolation and WFH intensity

**Figure 2:**
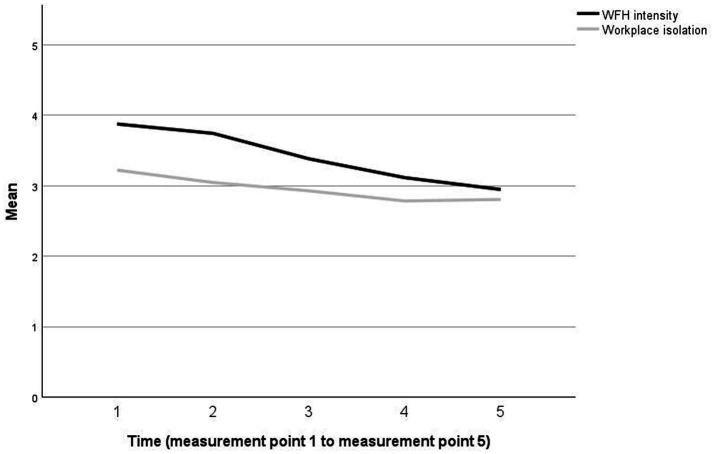
Trajectories of workplace isolation and WFH intensity over time (averaged across all participants).

The intraclass coefficient for our dependent variable workplace isolation was 0.65, indicating a 35% portion of within-person variance and justifying the use of a multilevel approach. In Hypothesis 1, we expected (1a) a negative nonlinear relationship between time and workplace isolation and (1b) an interaction effect of time and WFH intensity on workplace isolation. To determine the shape and growth parameters of the averaged sample trajectory, we first included a linear term of time as a predictor of workplace isolation and an interaction term of time and WFH intensity (model 1), then progressed with testing a quadratic (model 2) and cubic term of time (model 3). The results are displayed in Table 3. In model 1 no linear relationship between time and workplace isolation was found: *b* = −0.04, *SE* = 0.03, *p* = 0.176, but an interaction effect between time and WFH intensity on workplace isolation: *b* = −0.02, *SE* = 0.01, *p* < 0.01. Model 2 showed that in addition to the linear term, *b* = −0.46, *SE* = 0.12, *p* < 0.001, the quadratic term positively predicted workplace isolation: *b* = 0.07, *SE* = 0.02, *p* < 0.01. Additionally, we found an interaction effect of the quadratic term of time and WFH intensity: *b* = −0.01, *SE* = 0.01, *p* < 0.05. Model 3 revealed that none of the linear, quadratic or cubic terms of time nor any interaction effects predicted workplace isolation. Besides fixed effects, random effects were also tested in each model. For the first-order polynomial (linear trend, relationship between time and workplace isolation), the variance in intercepts across participants was: *b* = 0.73, *SE* = 0.10, *p* < 0.001, the variance in slopes across participants was: *b* = 0.01, *SE* = 0.00, *p* < 0.001, and a negative covariance of slopes and intercepts was seen: *b* = −0.29, *SE* = 0.08, *p* < 0.01. The comparison of models using chi-square statistics indicated that model 2 showed a relevant increase in -2LL, while model 3 did not, suggesting that model 2 described the best model fit. These statistical fit values (significance of the model comparison using -2LL, significance of fixed and random effects) indicate that both a linear trend (first-order polynomial) and a quadratic trend (second-order polynomial) best describe the data. The GCA found a negative linear and a positive quadratic relationship between time and workplace isolation. Accordingly, after a negative linear decrease, a flattening of the workplace isolation curve can be observed. Furthermore, the interaction between time and WFH intensity on workplace isolation seems to be relevant, suggesting that the effect of time on workplace isolation also varied with WFH intensity. This means that the change in workplace isolation is not only a function of time but is also affected by WFH intensity. H1a and H1b were thus supported. Figure 2 presents the averaged trajectories of workplace isolation and WFH intensity across all participants over 5 measurement points.

**Table 3:** Multilevel growth models for longitudinal change in workplace isolation and WFH intensity.

Effects	Model 1				Model 2				Model 3			
	*b*	*SE*	*p*	95% CI	*b*	*SE*	*p*	95% CI	*b*	*SE*	*p*	95% CI
Fixed effects												
Intercept	3.04	0.12	<0.001	2.81, 3.28	3.60	0.20	<0.001	3.21, 3.99	3.60	0.41	<0.001	2.80, 4.39
Time	−0.04	0.03	0.176	−0.09, 0.02	−0.46	0.12	< 0.001	−0.71, −0.21	−0.45	0.50	0.370	−1.42, 0.53
WFH intensity	0.07	0.03	0.026	0.01, 0.13	−0.04	0.05	0.389	−0.14, 0.06	−0.07	0.10	0.506	−0.27, 0.13
Time*WFH intensity	−0.02	0.01	0.008	−0.03, −0.01	0.06	0.03	0.072	−0.01, 0.13	0.10	0.13	0.452	−0.16, 0.35
Time^2^					0.07	0.02	0.001	0.03, 0.10	0.06	0.18	0.722	−0.29, 0.42
Time^2^*WFH intensity					−0.01	0.01	0.022	−0.02, −0.00	−0.03	0.05	0.564	−0.12, 0.07
Time^3^									−0.00	0.02	0.994	−0.04, 0.04
Time^3^*WFH intensity									0.00	0.01	0.731	−0.01, 0.01
Random effects												
Intercept π0	0.73	0.10	<0.001	0.56, 0.97	0.73	0.11	<0.001	0.55, 0.97	0.73	0.11	<0.001	0.55, 0.97
Time	0.01	0.00	<0.001	0.01, 0.02	0.01	0.00	<0.001	0.01, 0.02	0.01	0.00	<0.001	0.01, 0.02
WFH intensity	0.01	0.00	0.030	0.00, 0.03	0.01	0.00	0.032	0.00, 0.03	0.01	0.00	0.031	0.00, 0.03
Residual	0.36	0.01	<0.001	0.34, 0.39	0.36	0.01	<0.001	0.34, 0.39	0.36	0.01	<0.001	0.34,0.39
ARH1, rho	−0.29	0.08	0.001	−0.44, −0.12	−0.29	0.08	<0.001	−0.44, −0.12	−0.29	0.08	<0.001	−0.44, −0.12
Model fit indexes												
Parameters		9				11				13		
-2 log-likelihood		6144.05				6128.84**^,a^				6128.00		
𝜒^2^ statistic						15.21				0.85		
Degrees of freedom						2				2		

WFH = work from home. ^a^ Significant difference from the previous model. *p < 0.05, **p < 0.01, ***p < 0.001.

### 3.2 Distinct trajectories of workplace isolation and WFH intensity development

In Hypothesis 2, we expected four distinct trajectories of workplace isolation and WFH intensity development: (2a) two trajectories displaying high workplace isolation, one at low and one at high WFH intensity, and (2b) two trajectories displaying low workplace isolation, one at low and one at high WFH intensity. Using multiple statistical fit values as well as considering content decision criteria, six different LPA models (ranging from one to six profiles) were compared. We decided on the three-profile model for subsequent analyses due to significant *p*-values (*p* < 0.05) in all three likelihood ratio tests (LMRT, VLMRT and BLRT) as well as very high entropy and AvePP values indicating very good classification quality. Higher entropy or AvePP values indicate better fit. A perfect classification is at value 1, but reported cut-off value for entropy is 0.80 or higher. The same cut-off value is suggested as a result of the review by Spurk et al. (2020). In addition, ideally, the model with the lowest BIC value should be selected, as it represents the best fit (Spurk et al., 2020). Although the BIC value decreased with a higher number of profiles in the models in our analysis, the three-profile model still showed better fit values in all other statistical parameters (see Table 4).

**Table 4:** Latent profile analysis of workplace isolation and WFH intensity from T1 to T5.

No. of profiles	logL	Free parameters	BIC	LMRT	VLMRT	BLRT	Entropy	AvePP	Latent trajectory proportions
1	−8255.82	20	16636.42	─	─	─	─	─	512 (100%)
2	−7590.68	31	15374.74	0.000	0.000	0.000	0.92	0.98–0.98	177 (35%)/335 (65%)
3	−7040.32	42	14342.64	0.000	0.000	0.000	0.92	0.95–0.98	140 (27%)/190 (37%)/182 (36%)
4	−6876.60	53	14083.84	0.129	0.132	0.000	0.91	0.92–0.97	109 (21%)/149 (29%)/130 (25%)/124 (25%)
5	−6801.94	64	14003.13	0.070	0.071	1.000	0.93	0.00–0.97	78 (15%)/146 (29%)/180 (35%)/108 (21%)/0 (0%)
6	−6547.36	75	13562.600	0.166	0.170	0.000	0.92	0.91–0.96	63 (12%) / 103 (20%) / 103 (20%) / 48 (9%) / 79 (15%) / 116 (23%)

*N* = 512. logL = Log-likelihood. BIC = Bayesian Information Criterion. LMRT = Lo–Mendell–Rubin likelihood ratio test. VLMRT = Vuong-Lo–Mendell–Rubin likelihood ratio test. BLRT = bootstrapped likelihood ratio test. Entropy = Overall accuracy of classification. AvePP = average latent class posterior probabilities.

**Figure 3:**
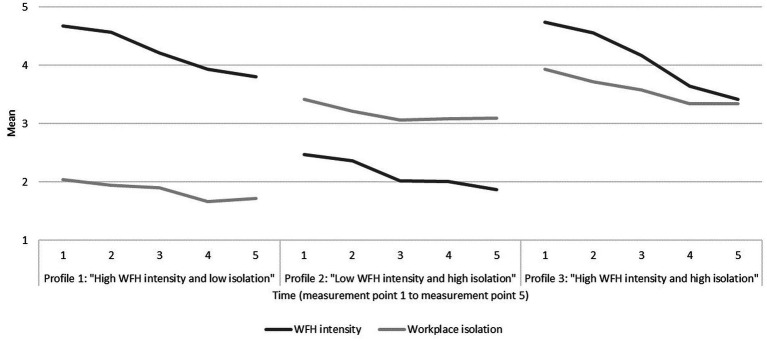
Sample means of workplace isolation and WFH intensity over time across profiles.

The three-profile model also showed a good distribution in terms of trajectory prevalence and ensured interpretability, as all three profiles were quantitatively distinct from each other (Spurk et al., 2020). Figure 3 displays the overall development of workplace isolation and WFH intensity across five measurement points for each profile. The first profile (*n* = 140, 27%), labelled “high WFH intensity and low isolation”, is characterized by high WFH intensity and low workplace isolation over time. Participants in this profile showed slight decreases in WFH intensity (mean T1 = 4.67, mean T5 = 3.81) as well as in workplace isolation over time (mean T1 = 2.04, mean T5 = 1.72). The second profile (*n* = 190, 37%) demonstrated nearly opposite characteristics to profile 1 and is therefore described as “low WFH intensity and high isolation”: Participants in profile 2 displayed slight decreases in low WFH intensity means over time (mean T1 = 2.47, mean T5 = 1.87) as well as in high workplace isolation means over time (mean T1 = 3.41, mean T5 = 3.10). The third profile (*n* = 182, 36%), labelled “high WFH intensity and high isolation”, is best described by participants working intensively from home over time, but also indicating high workplace isolation. Again, this profile demonstrated slight decreases in both variables over time (WFH intensity: mean T1 = 4.74, mean T5 = 3.41; workplace isolation: mean T1 = 3.93, mean T5 = 3.34).

In summary, there are two profiles characterized by high workplace isolation over time, with one profile displaying a high WFH intensity (profile 3) and the other profile displaying a low WFH intensity (profile 2). In contrast, profile 1 is characterized by low workplace isolation over time along with high WFH intensity. All profiles were similar in slopes of both variables but differed in their intercepts and were therefore quantitatively distinct from each other. Since the expected trajectory of low workplace isolation and low WFH intensity was not found in our data, H2 was partly supported.

### 3.3 Antecedents of profile membership: individual and social determinants

The LPA resulted in a new categorical variable in which each person was assigned to one of the three extracted trajectories (profile 1: “high WFH intensity and low isolation”, profile 2: “low WFH intensity and high isolation” and profile 3: “high WFH intensity and high isolation”) based on maximum fit. To examine predictors of profile membership, we conducted a multinomial logistic regression using our new categorial variable as dependent variable and predictor variables as independent variables.

In Hypothesis 3, we expected a positive association between SelfCare (H3a) and trajectory profiles that indicate low workplace isolation and high WFH intensity, and a negative association between extraversion (H3b) and those trajectory profiles. In Hypothesis 4, we expected a positive association between social norm, social support by colleagues, StaffCare and trajectory profiles that indicate low workplace isolation and high WFH intensity (H4a), and a negative association between communication difficulties and those trajectory profiles (H4b). Results are displayed in Table 5.

**Table 5:** Results of multinomial logistic regression analyses to predict profile membership.

Profile comparison	*b (SE)*	Wald	*p*	OR	95% CI for OR
Profile 2 vs. Profile 1					
Intercept	0.83 (1.70)	0.24	0.625		
Gender	0.07 (0.34)	0.04	0.830	1.08	[0.55, 2.11]
Age	−0.25 (0.02)	2.78	0.095	0.98	[0.95, 1.00]
Care for (grand) children while WFH	0.07 (0.15)	0.22	0.640	1.08	[0.79, 1.45]
Voluntariness of WFH	0.11 (0.13)	0.72	0.397	1.12	[0.87, 1.44]
SelfCare	−0.61 (0.35)	3.02	0.082	0.54	[0.27, 1.08]
Extraversion	0.46 (0.20)	5.25	0.022	1.58	[1.07, 2.33]
Social norm	−0.39 (0.14)	7.74	0.005	0.68	[0.52, 0.89]
Social support by colleagues	−0.36 (0.17)	4.73	0.030	0.70	[0.50, 0.97]
StaffCare	0.55 (0.23)	5.70	0.017	1.73	[1.10, 2.71]
Communication difficulties	0.72 (0.23)	10.17	0.001	2.06	[1.32, 3.21]
Profile 3 vs. Profile 1					
Intercept	−2.24 (1.58)	2.00	0.157		
Gender	0.18 (0.30)	0.34	0.559	1.19	[0.66, 2.17]
Age	−0.01 (0.01)	1.04	0.307	0.99	[0.96, 1.01]
Care for (grand) children while WFH	0.13 (0.14)	0.87	0.350	1.14	[0.87, 1.50]
Voluntariness of WFH	0.04 (0.11)	0.13	0.723	1.04	[0.84, 1.29]
SelfCare	−0.88 (0.31)	8.06	0.005	0.41	[0.22, 0.76]
Extraversion	0.42 (0.18)	5.53	0.019	1.52	[1.07, 2.14]
Social norm	0.32 (0.14)	5.30	0.021	1.38	[1.05, 1.82]
Social support by colleagues	−0.04 (0.16)	0.06	0.807	0.96	[0.71, 1.30]
StaffCare	0.53 (0.20)	6.80	0.009	1.70	[1.14, 2.53]
Communication difficulties	0.88 (0.21)	18.04	0.000	2.40	[1.60, 3.59]

*R*^2^ = 0.25 (Cox & Snell), 0.28 (Nagelkerke), 0.13 (McFadden). Model 𝜒^2^ (20) = 90.64, *p* < 0.001. *b* = regression coefficient, *SE* = standard error, OR = odds ratio, 95% CI = 95% confidence interval. StaffCare = health-oriented employee leadership, SelfCare = health-oriented self-leadership, WFH = work from home. All predictors and control variables were measured at T1. Degrees of freedom were 1 for all Wald statistics. Profile 1 = “High WFH intensity and low isolation”, profile 2 = “Low WFH intensity and high isolation”, profile 3 = “High WFH intensity and high isolation”.

Our final model explained a significant amount of original variability (𝜒^2^ [20] = 90.64, *p* < 0.001) and showed a good fit to our data according to Pearson test (𝜒^2^ [616] = 627.48, *p* = 0.365). The analysis results of the comparison of profile 2 (“low WFH intensity and high isolation”) with our reference category profile 1 (“high WFH intensity and low isolation”) indicated that extraversion, social norm, social support by colleagues, StaffCare and communication difficulties significantly predicted profile membership. Thereby, as social norm (*b* = −0.39, Wald 𝜒^2^[1] = 7.74, *p* < 0.01) and social support by colleagues (*b* = −0.36, Wald 𝜒^2^[1] = 4.73, *p* < 0.05) increased by one unit, the relative probability of belonging to profile 2 (compared to profile 1) decreased (change in the odds was 0.68 and 0.70, respectively). This means that social norm and social support by colleagues were positively associated with profile 1 in comparison to profile 2. Further, as extraversion (*b* = 0.46, Wald 𝜒^2^[1] = 5.25, *p* < 0.05), StaffCare (*b* = 0.55, Wald 𝜒^2^[1] = 5.70, *p* < 0.05) and communication difficulties (*b* = 0.72, Wald 𝜒^2^[1] = 10.17, *p* < 0.01) increased by a unit, the relative probability of belonging to profile 2 (compared to profile 1) increased (change in the odds was 1.58, 1.73, and 2.06 respectively). Extraversion, StaffCare and communication difficulties were therefore negatively related to profile 1 in comparison to profile 2.

The analysis results of the comparison of profile 3 (“high WFH intensity and high isolation”) with our reference category profile 1 (“high WFH intensity and low isolation”) indicated that SelfCare, extraversion, social norm, StaffCare and communication difficulties significantly predicted profile membership. As Selfcare (*b* = −0.88, Wald 𝜒2[1] = 8.06, *p* < 0.01) increased by one unit, the relative probability of belonging to profile 3 (compared to profile 1) decreased (change in the odds was 0.41). This means that SelfCare was positively associated with profile 1 in comparison to profile 3. Last, as extraversion (*b* = 0.42, Wald 𝜒^2^[1] = 5.53, *p* < 0.05), social norm (*b* = 0.32, Wald 𝜒^2^[1] = 5.30, *p* < 0.05), StaffCare (*b* = 0.53, Wald 𝜒^2^[1] = 6.80, *p* < 0.01) and communication difficulties (*b* = 0.88, Wald 𝜒^2^[1] = 18.04, *p* < 0.001), increased by one unit, the relative probability of belonging to profile 3 (compared to profile 1) increased (change in the odds was 1.52, 1.38, 1.70 and 2.40, respectively). Extraversion, social norm, StaffCare and communication difficulties were therefore negatively related to profile 1 in comparison to profile 3.

H3 was supported as SelfCare was positively and extraversion negatively associated with trajectory profile 1, indicating low workplace isolation and high WFH intensity. H4a was partly supported as only social support by colleagues was positively associated with trajectory profile 1, indicating low workplace isolation and high WFH intensity. Results on social norms were mixed: the comparison between profiles 2 and 1 revealed a positive association with profile 1, while the comparison between profiles 3 and 1 revealed a negative association with profile 1. Contrary to expectations, StaffCare was negatively associated with trajectory profile 1, indicating low workplace isolation and high WFH intensity. Interpretations of these results are presented in the discussion section. H4b was supported as communication difficulties were negatively associated with trajectory profile 1, indicating low workplace isolation and high WFH intensity.

### 3.4 Consequences of profile membership: health-and work-related outcomes

To examine differences of profile membership on outcomes, we performed ANOVAs with post hoc tests. We used our categorial variable, profile membership, as independent variable and outcome variables as dependent variables. In Hypothesis 5, we expected lower levels of psychological strain (H5a), and higher levels of commitment (H5b) and self-rated performance (H5c) for trajectory profiles indicating low workplace isolation and high WFH intensity. Since profile 1 in our analysis is characterized by low workplace isolation and high WFH intensity, this profile was compared with both other profiles in the analysis.

Results revealed no significant differences between profiles on levels of psychological strain, *F*(2, 509) = 2.15, *p* = 0.117. H5a was therefore rejected. Possible interpretations of these findings are outlined in the discussion.

**Figure 4:**
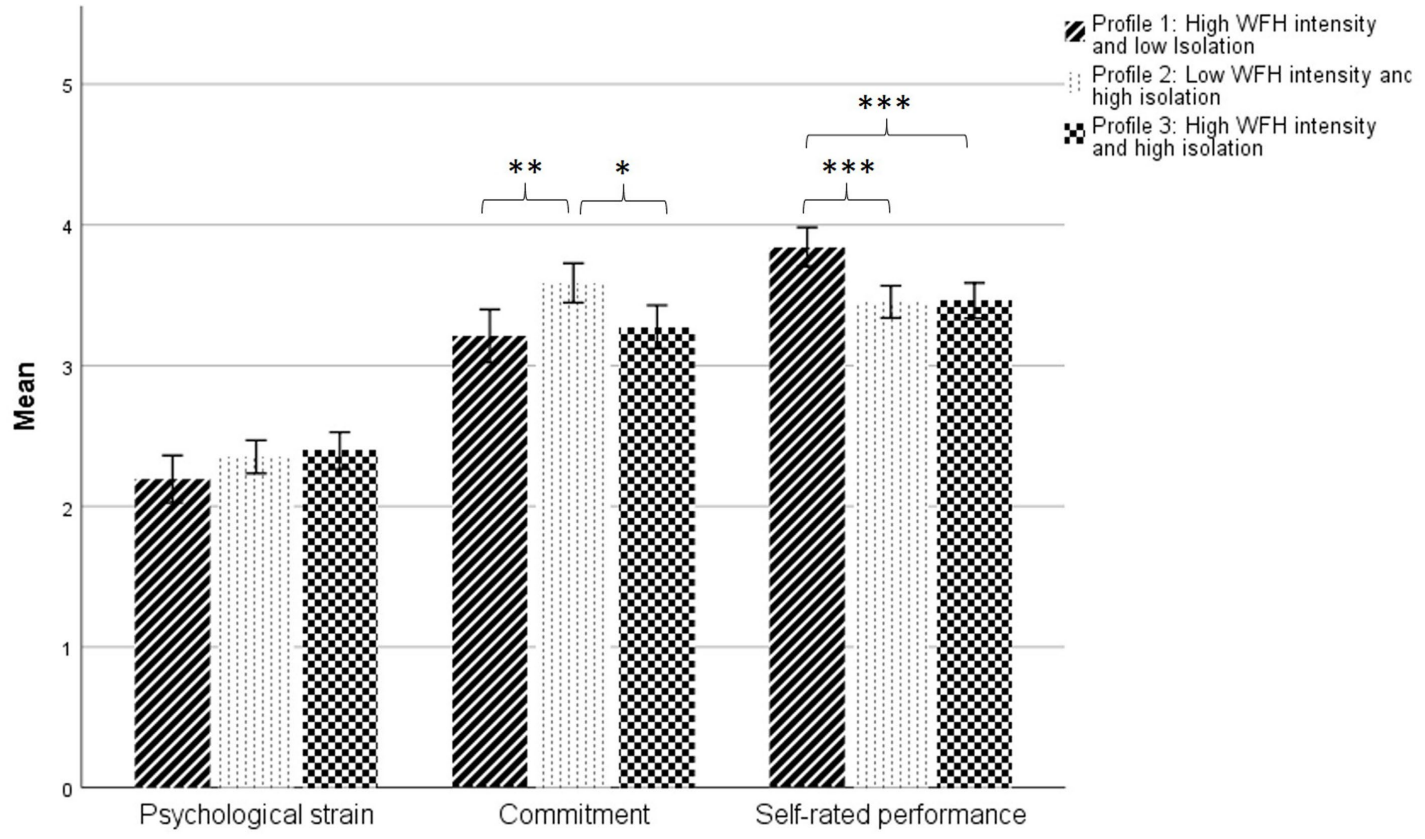
Post hoc comparisons between profiles.Note: Error bars are 95% confidence intervals. Verbal anchors for psychological strain: 1 (*never*) to 5 (*almost always*), for commitment: (*does not apply at all*) to 5 (*totally applies*), for performance: 1 (*sufficient*) to 5 (*excellent*). Significant differences between profiles: *p < 0.05, **p < 0.01, ***p < 0.001.

Testing H5b, results displayed significant differences between profiles on levels of commitment, *F*(2, 509) = 6.47, *p* < 0.01, ω^2^ = 0.021, indicating a small effect size. Due to non-equal group sample sizes and homogeneity of variance, Hochberg’s GT2 post hoc analysis was used and revealed a significant difference between commitment levels of profiles 1 (“high WFH intensity and low isolation”) and 2 (“low WFH intensity and high isolation”), indicating lower commitment levels in profile 1 (M_Profile1_ = 3.21 vs. M_Profile2_ = 3.59; mean diff. = − 0.38, *p* < 0.01, 95%-CI [−0.66, −0.10], see Figure 4), but no significant difference between profiles 1 (“high WFH intensity and low isolation”) and 3 (“high WFH intensity and high isolation”; M_Profile1_ = 3.21 vs. M_Profile3_ = 3.27; mean diff. = − 0.06, *p* = 0.929, 95%-CI [−0.35, 0.22]). Since only one of the two group comparisons was tested significantly, H5b was rejected. Supplementary analysis identified a significant difference between commitment levels of profiles 2 (“low WFH intensity and high isolation”) and 3 (“high WFH intensity and high isolation”; M_Profile2_ = 3.59 vs. M_Profile3_ = 3.27; mean diff. = 0.31, *p* < 0.05, 95%-CI [0.05, 0.57]), indicating higher commitment levels in profile 2. Contrary to expectations, higher commitment values were not associated with the profile displaying low workplace isolation and high WFH intensity (i.e., profile 1), but instead with the profile displaying high workplace isolation and low WFH intensity (i.e., profile 2).

Last, testing H5c, results revealed significant differences between profiles on levels of self-rated performance, *F*(2, 509) = 10.93, *p* < 0.001, ω^2^ = 0.04, also indicating a small effect size. Again, Hochberg’s GT2 post hoc analysis revealed significant differences between performance levels of profiles 1 (“high WFH intensity and low isolation”) and 2 (“low WFH intensity and high isolation”; M_Profile1_ = 3.84 vs. M_Profile2_ = 3.45; mean diff. = 0.39, *p* < 0.001, 95%-CI [0.17, 0.61]) and between profiles 1 (“high WFH intensity and low isolation”) and 3 (“high WFH intensity and high isolation”; M_Profile1_ = 3.84 vs. M_Profile3_ = 3.46; mean diff. = 0.38, *p* < 0.001, 95%-CI [0.16, 0.61]), indicating higher self-rated performance levels in profile 1 in comparison to both other profiles 2 and 3. H5c was supported.

## 4 Discussion

Previous longitudinal studies on isolation perceptions during the pandemic were primarily conducted to analyze loneliness in the general population. Using both a variable-and person-oriented approach, this study was the first to analyze the growth curve of workplace isolation and WFH intensity of employees over the course of the pandemic, to identify distinct trajectories (subgroups) and determine antecedents and consequences of these subgroups with different trajectories.

### 4.1 Multilevel growth curve analysis for changes in workplace isolation and WFH intensity

Our multilevel GCA found that changes in workplace isolation were best predicted by a linear and quadratic trend, indicating a nonlinear relationship between time and workplace isolation. Accordingly, it was found that participants’ workplace isolation first decreased over the course of the pandemic and then plateaued. Given that time predicted the trajectory of workplace isolation and thus a general trend over time was identified in our sample, we may conclude that the changing conditions during the pandemic had an overall impact on the perception of workplace isolation. Although there are no comparable studies on workplace isolation during the pandemic, heterogeneous research on loneliness (Bu et al., 2020; Buecker et al., 2020; Entringer and Kröger, 2020; Killgore et al., 2020; Luchetti et al., 2020; Caro et al., 2022; Hettich et al., 2022; Benke et al., 2023) suggests that other factors might also affect trajectories of workplace isolation. Consistent with literature linking WFH intensity to workplace isolation (Sahai et al., 2020; Gajendran et al., 2024; Rudolph and Zacher, 2024), our findings suggest that the effect of time on workplace isolation varies with WFH intensity. Thus, employees’ workplace isolation over time was influenced by their WFH intensity during the pandemic. We may conclude that the trajectory of workplace isolation can be explained by both external pandemic conditions (effect of time) as well as individual variation (interaction effect of WFH intensity). WFH intensity seems to be a relevant variable in analyzing workplace isolation in remote collaboration, also presumably independent of pandemic conditions. In addition, possible explanations for the stabilization of the workplace isolation trajectory may include organizational adjustments or improved remote work practices during the pandemic (De Vincenzi et al., 2022).

### 4.2 Distinct trajectories of workplace isolation and WFH intensity development

Similar to our expectations, our LPA revealed three distinct trajectories: profile 1 “high WFH intensity and low isolation”, profile 2 “low WFH intensity and high isolation”, and profile 3 “high WFH intensity and high isolation”. Interestingly, a profile with low workplace isolation and WFH intensity was not found. The slopes of both variables were similarly decreasing across all three profiles, indicating quantitative but not qualitative differences. Quantitative differences refer to level differences between profiles, qualitative differences to shape differences between profiles in LPA (Spurk et al., 2020). Our study found that most employees experienced high workplace isolation (profile 2 and 3, about 73%), though half of them worked from home either very frequently or little. This is in contrast to previous studies which have shown lower proportions of high loneliness or low workplace sense of community in general populations: 5.0% (Laham et al., 2021), 14.3% (Bu et al., 2020), 15.8% (Graham et al., 2023), 8.0% (severe) and 28.0% (high; Caro et al., 2022). Additionally, the slopes of these trajectories differed from our results: in two studies, high loneliness levels increased over the course of the pandemic (Bu et al., 2020; Laham et al., 2021); in two other studies, they remained stable (Caro et al., 2022; Graham et al., 2023). This comparison to previous longitudinal studies during the pandemic is limited, since these studies mostly investigated loneliness (i.e., subjective perceptions of qualitative or quantitative deficiencies in social relationships; Russell et al., 1984) in general populations, whereas workplace isolation in our study specifically refers to employees’ subjective perception of social isolation from others at work (Marshall et al., 2007; Golden et al., 2008).

Overall, all three profiles displayed decreasing slopes over time (consistent with the averaged trajectory in our GCA). This suggests that contextual conditions influenced WFH intensity (presumably due to a decline in national regulations regarding containment measures and better control of the spread of the virus during the pandemic) and, in turn, affected workplace isolation. Although, to our knowledge, there is hardly any research with LPAs using two variables, our results demonstrate that this methodological approach yields additional informative value. Both our GCA and LPA results indicate that WFH intensity is a relevant covarying variable for identifying distinct subgroups of workplace isolation.

### 4.3 Antecedents of profile membership: individual and social determinants

In line with our expectations, our results on *individual determinants* showed that SelfCare and extraversion predicted profile membership. Similar to previous research (Krick et al., 2024a), SelfCare was positively associated with profile 1 (“high WFH intensity and low isolation”) in comparison to profile 3 (“high WFH intensity and high isolation”). This suggests SelfCare to be a protective factor against high workplace isolation at high WFH intensity. Further, extraversion was negatively associated with profile 1 (“high WFH intensity and low isolation”) in comparison to both other profiles, indicating that extraverted people feel more isolated in remote teams irrespective of own WFH intensity. This result is consistent with previous research findings, as this Big Five trait was generally associated with higher engagement in social activities (Lucas et al., 2008). Links between extraversion and preference for teleworking (Gavoille and Hazans, 2022) or higher levels of missing colleagues (Demerouti et al., 2014; Langvik et al., 2021) were also identified in the context of remote working.

Regarding *social determinants*, our study identified social norm, social support by colleagues, StaffCare, and communication difficulties as predictors of profile membership. As expected, communication difficulties were negatively associated with profile 1 (“high WFH intensity and low isolation”) in comparison to both other profiles, suggesting that communication difficulties in remote collaboration facilitate workplace isolation regardless of WFH intensity. Similarly, previous studies also referred to communication challenges in remote collaboration (Marshall et al., 2007; Tautz et al., 2022; Tautz et al., 2024), as well as conducive impacts of communication quantity on isolation (Golden et al., 2008; Van Zoonen and Sivunen, 2022; Wong et al., 2022). Furthermore, in line with our expectations and previous research (Marshall et al., 2007; Sahai et al., 2020), social support by colleagues was positively associated with profile 1 (“high WFH intensity and low isolation”) compared to profile 2 (“low WFH intensity and high isolation”), indicating that it is a job resource that mitigates isolation. Moreover, our results on social norm were mixed. It was negatively associated with profile 1 (“high WFH intensity and low isolation”) in comparison to profile 3 (“high WFH intensity and high isolation”), which contrary to our expectations indicates higher isolation when many colleagues frequently WFH. However, social norm was positively associated with profile 1 (“high WFH intensity and low isolation”) in comparison to profile 2 (“low WFH intensity and high isolation”), suggesting that employees are more likely to WFH more frequently when the social norm is also high (i.e., a high level of WFH intensity in the team or department). This last result is in line with previous research, as a study found that teleworking colleagues had a stronger influence on telework adoption than non-teleworking colleagues (Scott et al., 2012). Since there may exist other motives for choosing to WFH, e.g., due to job requirements, efficiency or work-life balance reasons (Vanderstukken et al., 2022), the role of social norm on workplace isolation in WFH remains unclear. In addition, other contextual factors such as team cohesion or informal workplace culture may shape the perceived impact of social norms on workplace isolation in remote contexts (Sahai et al., 2020). Surprisingly, StaffCare was negatively associated with profile 1 (“high WFH intensity and low isolation”) in comparison to both other profiles. This is in contrast to our expectations and previous research, which pointed to a mitigating role of leadership on employees’ isolation perceptions in remote collaboration (Efimov et al., 2022) and to StaffCare’s beneficial influence on employee outcomes when WFH (Klebe and Felfe, 2023; Krick et al., 2023; Sachse and Schanz, 2023). It is possible that employees in this study felt more isolated when they perceived an overall high level of StaffCare, as they missed their leader’s positive influence when WFH (given that our workplace isolation variable was only measured for WFH context). Another possible explanation for this link may be that StaffCare is more effective in face-to-face settings and its impact is less perceptible in remote contexts (Klebe and Felfe, 2023). Digital communication could dilute the visibility of leadership behaviors, which may explain the weaker effect of StaffCare on perceived isolation when working from home.

Reflecting upon our theoretical framework, the JD-R and COR theories, we may conclude that employees with sufficient resources (e.g., high SelfCare or social support from colleagues) are more likely to gain resources or are more resistant to resource losses in the future, e.g., experience lower workplace isolation in remote collaboration. Conversely, employees facing job demands (such as communication difficulties) may struggle more with additional demands in remote collaboration due to pandemic, leading to higher isolation.

### 4.4 Consequences of profile membership: health-and work-related outcomes

Contrary to expectations and previous research (Sahai et al., 2020; Van Zoonen and Sivunen, 2022), there were no significant differences in psychological strain levels between profiles. Accordingly, psychological strain was probably independent of workplace isolation perceptions and WFH intensity. Further, contrary to our expectations, our results did not indicate higher commitment levels in profile 1 (“high WFH intensity and low isolation”). Instead, highest commitment levels were found for profile 2 (“low WFH intensity and high isolation”). Significant differences between profile 2 and both other profiles suggest that high WFH intensity may undermine commitment (since profile 2 is the only subgroup to display low WFH intensity over the course of the pandemic). In contrast, previous research pointed to a negative relationship between workplace isolation and affective commitment (Sahai et al., 2020). A recent meta-analysis referred to a detrimental effect of isolation perceptions as mediator in the relationship between WFH intensity and organizational commitment (Gajendran et al., 2024). In addition, alternative explanations for the findings on psychological strain and commitment are also possible, e.g., other variables such as organizational climate or role clarity may influence these relationships. Finally, in line with expectations, our results found highest levels of self-rated performance in profile 1 (“high WFH intensity and low isolation”) in comparison to both other profiles. This suggests that workplace isolation may be more decisive for self-rated performance than WFH intensity, since profile 1 is the only subgroup to display low workplace isolation over the course of the pandemic. Previous research also indicated a negative influence of workplace isolation on the relationship between remote work intensity and performance (Gajendran et al., 2024).

### 4.5 Strengths and limitations

Our study demonstrates several strengths. It contributes to research and practice by using a comprehensive, longitudinal data set and a novel approach by combining variable- and person-oriented methods. Thereby, we gained initial and holistic insights into between- and within-effects of workplace isolation and WFH intensity as well as into relevant antecedents and consequences of subgroups. Applying a longitudinal LPA with two variables is rather novel, but this methodological approach yielded additional informative value and enhanced our understanding of individual differences in the development of workplace isolation and WFH intensity.

Yet this study has some limitations. First, it was not always possible to ensure equal intervals between measurement times, although no considerable differences were found between adjacent values. Second, there are limitations with respect to the use of self-developed single-item scales. This is a common practice in large-scale surveys for reasons of research economy and for reasons of simpler and more comprehensible responding by study participants. If no validated short or single-item instruments are available in previous research as well as the constructs used are unidimensional, clearly defined and narrow in scope, their use is acceptable. Nevertheless, there is a potential limitation regarding measurement reliability and validity (Allen et al., 2022). For the same reasons of comprehensibility and consistency of the items for study participants, the verbal anchors of the scales used were standardized. Third, like many other methods, the LPA method is an approximation of reality, in which the model that best described the data (based on clear statistical fit values and substantive decision criteria; Spurk et al., 2020) was selected. Nevertheless, it must be pointed out that the identified profiles depend on our theory-driven selection of variables. Therefore, when rerunning an LPA on workplace isolation of remote workers with additional or different variables, other results may be obtained. Fourth, self-selection or self-report biases, e.g., social desirability or misjudgment, cannot be ruled out. Past research demonstrated discrepancies in self-other ratings, e.g., in leadership or performance (Heidemeier and Moser, 2009; Lee and Carpenter, 2018). Fifth, the dynamic course of the pandemic as the context of our study needs to be considered when interpreting the results. It remains open if similar results would emerge in non-pandemic contexts or post-implementation of virtual/hybrid teamwork. Sixth, although a large longitudinal sample was collected, potential limitations remain due to the geographical restriction to Germany. In this regard, the results on workplace isolation perceptions may also be influenced by cultural context (in contrast to non-European countries, for example), potentially limiting generalizability. Last, there is limited research on longitudinal studies of workplace isolation, reducing comparability of present results with previous research.

### 4.6 Implications for future research

Based on this study’s limitations, future research directions were derived. According to current state of research, there are no standardized definitions and thus different measurement instruments for assessing both workplace isolation and WFH intensity (Marshall et al., 2007; Sahai et al., 2020; De Vincenzi et al., 2022). Future research should conduct studies on the theoretical foundation, construct development and validation, e.g., a recent study on the team perceived virtuality scale (Handke et al., 2024). Thus, future efforts should aim for standardization of definitions and instruments or for context-specific operationalizations. Moreover, recommendations include conducting longitudinal GCA and LPA analyses of workplace isolation and WFH intensity outside a pandemic context to determine if these variables remain covarying. Such analyses are also recommended for interventional studies, e.g., to evaluate the implementation of virtual or hybrid teamwork in organizations. Further, research should explore workplace isolation with other potentially covarying variables (like WFH intensity) within longitudinal LPA. More generally, it is recommended for future studies to explore the LPA method with more than one variable or interaction effects. Due to mixed findings on social norm and unexpected results on StaffCare, future research should further explore the role of social norm and leadership in remote work in relation to workplace isolation. In addition to individual and social determinants, other factors at the organizational or workplace level (e.g., organizational climate or ICT demands) may also play a role in the development of workplace isolation when WFH and should therefore be included in future research. Overall, current research highlights the fact that many research gaps still exist on the antecedents and outcomes of workplace isolation, which is gaining relevance in light of past and current trend towards WFH (Sahai et al., 2020). In this regard, future research should investigate whether workplace isolation is a relevant predictor of self-rated performance, as assumed in our study. Future studies should also examine differences between employee groups, e.g., at varying hierarchical levels, and analyze self-other ratings in dyads (leader and employee) or teams (leader and team members), which are currently underexplored but increasingly relevant. Screening for illnesses like depression prior to study participation may also be considered.

### 4.7 Implications for practice

The results provide important practical implications. When implementing or increasing remote work, organizations should adapt working conditions to mitigate the negative impact of workplace isolation on employee outcomes (see also Gajendran et al., 2024). Especially in times of crisis, such as pandemics, which cause disruptive changes in the workplace, organizations should consider that employees may differ in their experiences, e.g., isolation perceptions both during high and low WFH intensity. Participatory approaches and regular employee surveys that address diverse needs are essential. This knowledge allows organizations to tailor their support strategies by strengthening relevant resources (e.g., SelfCare, social support by colleagues) and reducing demands (e.g., communication difficulties). In view of the fact that workplace isolation may have a relevant impact on self-perceived performance, organizations should consider prevention in this context.

At the *employee level*, organizations should provide SelfCare trainings, helping employees understand and meet their needs in remote collaboration (e.g., an increased need for social exchange for those with high levels in extraversion). For the HoL model in particular, one such intervention is the GoFüKo training program, which focuses on developing SelfCare and StaffCare competencies (Krick and Felfe, 2024b). Mindfulness-based interventions have also shown to improve SelfCare (Krick et al., 2018; Krick and Felfe, 2020, 2024a).

At the *leadership level*, training for leaders in remote collaboration should cover discussing employees’ individual needs and challenges in regular one-on-one meetings, detecting early warning signs of mental illnesses and fostering social exchange (formal and informal meetings, both in person and remotely). Further, leaders should learn how to strengthen social relationships within the team and their team identity and adapt their StaffCare behavior in digital communication. While leadership interventions (e.g., health-oriented leadership interventions) have shown positive effects in traditional, face-to-face work settings (Stuber et al., 2021; Dannheim et al., 2022), there are hardly any such studies for the remote context. When implementing training for leaders in remote work, organizations should pay attention to offering low-threshold and feasible training (e.g., scalable practices or digital tools). An initial tool specifically addressing leaders to overcome specific challenges in remote work is the digital tool DigiLAP (Krick et al., 2024b).

In order for behavioral prevention approaches to be successfully implemented, organizations need to adequately address working conditions at an *organizational level*. Therefore, working conditions in virtual or hybrid collaboration should be established that allow employees to apply SelfCare behavior, e.g., clear agreements on availability times or establishment of digital meeting etiquette. To prevent communication difficulties, teams should be able to receive internal mediators or external experts for team consultation. Further, organizations should promote team collaboration by offering team development activities and facilitating informal exchanges in person. Finally, organizations have to ensure adequate technical preconditions for remote collaboration and offer IT support services (Efimov et al., 2024a).

## 5 Conclusion

While previous research has primarily focused on studying loneliness in the general population during the pandemic, this study offers a novel contribution by analyzing workplace isolation in relation to WFH intensity over time. By combining variable-and person-oriented approaches, this study identifies a general trend and subgroups with distinct trajectories, their antecedents and consequences. We found that workplace isolation decreased during the pandemic, then plateaued, and covaried with WFH intensity. Three distinct profiles among participants differed in their initial levels of isolation and WFH intensity, though they followed similar downward trends over time, suggesting that pandemic’s contextual conditions influenced workplace isolation and WFH intensity development. By identifying relevant resources (e.g., SelfCare, social support by colleagues) and demands (e.g., communication difficulties), important implications for practice for reducing workplace isolation were derived. Recommendations for organizations include behavioral and structural prevention measures for employees and leaders in remote collaboration, e.g., SelfCare and leadership trainings to promote social relationships and prevent communication difficulties. In view of remaining theoretical, methodological and empirical research gaps, future research is encouraged to develop theoretically grounded and psychometrically validated instruments to assess workplace isolation, especially in light of evolving work arrangements such as hybrid and fully remote models.

## Data Availability

The datasets presented in this article are not readily available because of privacy restrictions. Requests to access the datasets should be directed to JF, felfe@hsu-hh.de.
