# Supplementary material for: When do employees feel isolated when working from home? Longitudinal trajectories, antecedents and outcomes of workplace isolation during the COVID-19 pandemic
Source: Front Psychol. 2025 Jul 8;16:1601214. doi: 10.3389/fpsyg.2025.1601214 (PMC12279890; doi:10.3389/fpsyg.2025.1601214)
Supplement: Supplementary file 1 [file Data_Sheet_1.pdf]

Supplementary material 1. Overview of variables used in the study.

	T1	T2	T3	T4	T5
Main variables					
Workplace isolation	x	x	x	x	x
WFH intensity	x	x	x	x	x
Predictors					
SelfCare	x				
Extraversion	x				
Social norm	x				
Social support by colleagues	x				
StaffCare	x				
Communication difficulties	x				
Outcomes					
Psychological strain					x
Affective organizational commitment					x
Self-rated performance					x
n	512	512	512	512	512
